# Allopurinol for Secondary Prevention in Patients with Cardiovascular Disease: A Systematic Review and Meta-Analysis of Randomized Controlled Trials

**DOI:** 10.3390/jcdd10090379

**Published:** 2023-09-04

**Authors:** Yuyang Ye, Guangzhi Liao, Ting Liu, Xinru Hu, Xuefeng Chen, Lin Bai, Yong Peng

**Affiliations:** 1 Department of Cardiology, West China Hospital, Sichuan University, 37 Guoxue Street, Chengdu 610041, China; yeyuyangcd@163.com (Y.Y.); soleil0726@163.com (G.L.); liutingmed@163.com (T.L.); cxfhxlc2017@163.com (X.C.); doctor_bai@163.com (L.B.); 2 School of Medicine, Zhengzhou University, Zhengzhou 450052, China; huxinrudoct@163.com

**Keywords:** allopurinol, cardiovascular disease, cardiovascular outcomes, febuxostat

## Abstract

Background: The effects of allopurinol in patients with cardiovascular disease are not well defined; therefore, the latest evidence is summarized in this study. Methods: PubMed, Embase, Cochrane Library, and ClinicalTrials.gov databases were searched for randomized controlled trials (RCTs) of allopurinol in patients with cardiovascular disease published up to 11 February 2023. The primary outcome was cardiovascular death. Results: We combined the results of 21 RCTs that included 22,806 patients. Compared to placebo/usual care, allopurinol treatment was not associated with a significant reduction in cardiovascular death (RR 0.60; 95% CI 0.33–1.11) or all-cause death (RR 0.90; 95% CI 0.72–1.12). However, evidence from earlier trials and studies with small sample sizes indicated that allopurinol might confer a protective effect in decreasing cardiovascular death (RR 0.34; 95% CI 0.15–0.76) across patients undergoing coronary artery bypass grafting (CABG) or having acute coronary syndrome (ACS). In comparisons between allopurinol and febuxostat, we observed no difference in cardiovascular death (RR 0.92; 95% CI 0.69–1.24) or all-cause death (RR 1.02; 95% CI 0.75–1.38). Conclusion: Allopurinol could not reduce cardiovascular (CV) death or major adverse CV outcomes significantly in patients with existing cardiovascular diseases. Given the limitations of the original studies, the potential advantages of allopurinol observed in patients undergoing CABG or presenting with ACS necessitate further confirmation through subsequent RCTs. In the comparisons between allopurinol and febuxostat, our analysis failed to uncover any marked superiority of allopurinol in reducing the risk of adverse cardiovascular incidents.

## 1. Introduction

Despite guideline-recommended pharmacotherapy for populations with cardiovascular disease (CVD), the mortality and incidence of adverse cardiovascular events remain unacceptably high [1]. The substantial disease burden and poor prognosis necessitate the improvement of therapeutic interventions. In recent decades, considerable studies have demonstrated the significant association between serum uric acid (SUA) levels and cardiovascular disease [2]. Even after adjustment for other traditional cardiovascular risk factors, SUA levels persist as a robust predictor of adverse cardiovascular events in CVD, including hypertension, heart failure, coronary artery disease (CAD), and atrial fibrillation [3,4,5,6]. Subsequently, allopurinol, a xanthine oxidase inhibitor (XOI) licensed for the prophylaxis of gout or symptomatic hyperuricemia, has been proposed as a potentially effective medication and is associated with the reduction in adverse cardiovascular endpoints according to a growing body of evidence [7]. In addition to its established efficacy in decreasing uric acid levels, the antioxidant capacity of allopurinol in cardiovascular disease has emerged as an increasingly prominent topic in recent years.

Despite the potential of allopurinol for patients with CVD, evidence of clinical benefit is still lacking [8]. Higgins et al. summarized the potential therapeutic role of XOI in cardiovascular disease and showed its positive association with endothelial function and circulating markers of oxidative stress in patients with CVD [9]. Nevertheless, some crucial trials were not identified in that study, and quantitative analyses related to critical clinical endpoints, including death and adverse cardiovascular outcomes, were omitted [10]. In recent years, several large RCTs, such as FAST and ALL-HEART, have been published and reported mortality and cardiovascular outcomes in patients randomly assigned to allopurinol, febuxostat, or placebo/usual-care management [11,12]. In light of these developments, we designed this study to explore the role of allopurinol in the secondary prevention of CVD by systematically reviewing and updating the latest evidence from randomized controlled trials.

## 2. Materials and Methods

### 2.1. Study Design

This study has been registered on PROSPERO (CRD42023397268). We followed the Preferred Reporting Items for Systematic Reviews and Meta-Analysis statements [13] while performing and reporting this work. All data used in this study were extracted from individual studies.

### 2.2. Search Strategy

A search of all RCTs that compared allopurinol to a placebo or a non-allopurinol condition in patients with cardiovascular disease was conducted by two reviewers (Ye Y. and Liao G.) independently. PubMed, Embase, Cochrane Library, and ClinicalTrials.gov databases were searched to retrieve studies published from inception to 11 February 2023 without any restrictions on language or publication date. Primary search terms used were ‘allopurinol’, ‘myocardial ischaemia’, ‘coronary artery disease’, and ‘randomized controlled trial’, encompassing their respective subheadings and synonyms. The full search strategy is detailed in the Supplementary Materials. In addition, a snowball method was used to find potentially missed eligible studies from the references of key reviews and included articles. Discrepancies were discussed with a third senior author (Peng Y.) to reach a consensus.

### 2.3. Eligible Study Criteria and Endpoints of Interest

Eligible studies met the following criteria. (1) The patients enrolled were adults older than 18 years living with pre-existing cardiovascular disease at baseline. The definition of CVD consists of coronary artery disease, ischemic cardiomyopathy, hypertension, and heart failure. (2) Intervention: Allopurinol treatment, regardless of the dose used. (3) Comparative interventions: Placebo or febuxostat treatment. We deemed allopurinol versus placebo/usual care as the main comparison and allopurinol versus febuxostat as the most clinically relevant active comparison. (4) Outcomes: Only studies that provided outcome data of interest were included in this review. The primary outcome was cardiovascular death, and the secondary outcomes were the major cardiovascular event and its components, including all-cause death, myocardial infarction, and stroke, in a short (<30 days), medium-term (30 days to 1 year), and long-term (≥1 year) follow-up. (5) Study design: Only randomized controlled trials were included in this review. Studies not written in English or not peer-reviewed and registries with overlapping populations were excluded.

### 2.4. Data Collection and Extraction

The records identified from databases were exported to EndNote (version 20, Clarivate Analytics), and then duplicates were removed. The titles and abstracts of the records were screened by two independent reviewers (Ye Y. and Liao G.) for eligibility using the predetermined selection criteria. Full texts of all potentially eligible studies were then independently studied by two reviewers (Ye Y. and Liao G.) to determine the final selection. Discrepancies were discussed with a third senior author (Peng Y.) to reach a consensus. Two reviewers (Ye Y. and Liao G.) in parallel independently extracted and cross-checked the data using a predefined form. The following data were extracted from eligible studies: (1) the number of participants and their composition by age and sex; (2) CVD diagnoses; (3) follow-up time; (4) baseline comorbidities; (5) outcome data. Discrepancies were resolved through discussion.

### 2.5. Risk of Bias Assessment

Cochrane Collaboration’s revised Risk-of-Bias tool (RoB 2.0) [14] was used to assess the quality of RCTs by two independent reviewers (Ye Y. and Liao G.), which includes five domains for risk of bias: the randomization process, deviations from intended interventions, missing outcome data, measurement of the outcome, and selection of the reported results. Discrepancies were resolved through discussion.

### 2.6. Data Synthesis and Analysis

Pooled relative risks (RR) with 95% confidence intervals were calculated utilizing a random-effects model with the DerSimonian–Laird method. Between-study heterogeneity was assessed by calculating Higgins and Thompson’s I^2^ statistic, in which heterogeneity was considered substantial if I^2^ was over 50%. Where 10 or more studies were identified, we used funnel plots with the trim and fill method and Egger’s test to assess publication bias. In addition, subgroup analyses were conducted to investigate the influence of follow-up time, types of cardiovascular diseases, and history of hyperuricemia or gout on the results. Finally, sensitivity analysis was performed to explore the underlying causes of heterogeneity. In all tests, a two-tailed *p*-value <0.05 was considered statistically significant. All statistical analyses were performed using R software (URL http://www.R-project.org/ (accessed on 10 April 2023) version 4.2.2).

## 3. Results

### 3.1. Study Characteristics and Quality

**Figure 1:**
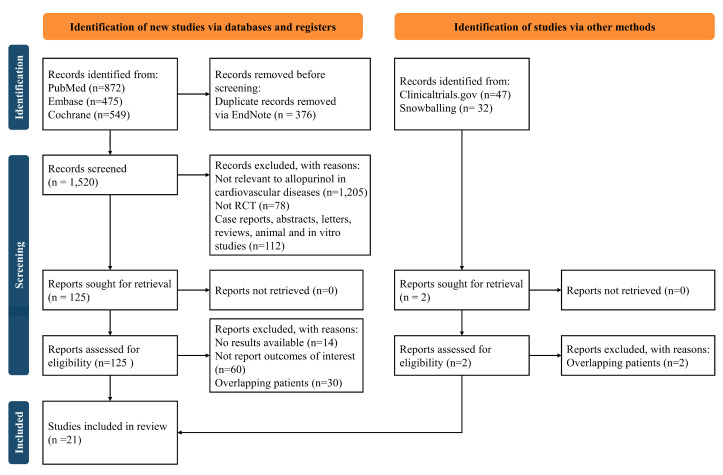
Flowchart of study selection.

**Figure 2:**
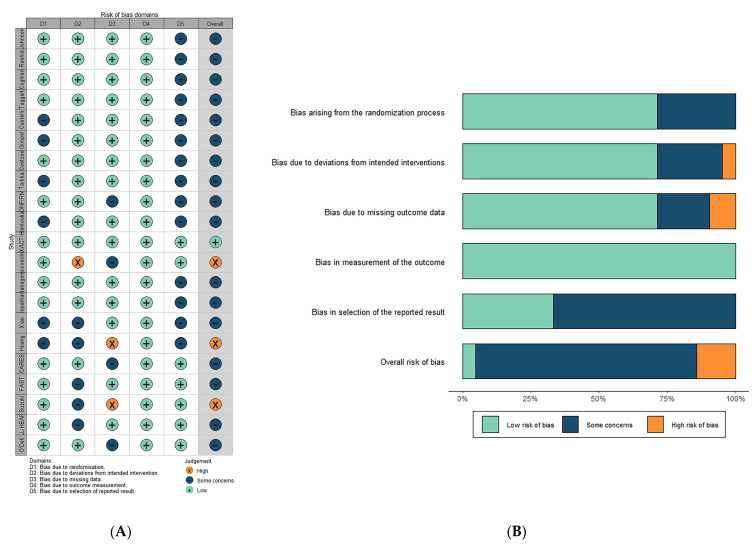
Risk of bias: (**A**) the risk of bias graph and (**B**) the risk of bias summary.

Figure 1 details the PRISMA systematic review flowchart. After review, a total of 21 eligible randomized controlled trials were included in this study, with Table 1 and Table S1 (Supplementary Materials) providing the key characteristics and the baseline characteristics of patients enrolled, respectively. A total of 15 trials consisting of 6955 patients and 6 studies comprising 15,851 subjects compared the effects of allopurinol to placebo/usual care and febuxostat, respectively. Among all the participants, the mean age was 66.8 years, and the mean female proportion was 16.5%. In comparison between allopurinol treatment and placebo/usual care, only one study was conducted on those with CVD and hyperuricemia or gout [15], while in comparison between allopurinol and febuxostat treatment, all six studies were conducted on patients with CVD and hyperuricemia or gout [11,16,17,18,19,20]. Figure 2 and Table S2 (Supplementary Materials) present the risk of bias of all the included studies, indicating that only three trials were classified as ‘high risk of bias’ [20,21,22].

### 3.2. Comparison 1. Allopurinol versus Placebo/Usual Care

#### 3.2.1. Cardiovascular Death

**Figure 3:**
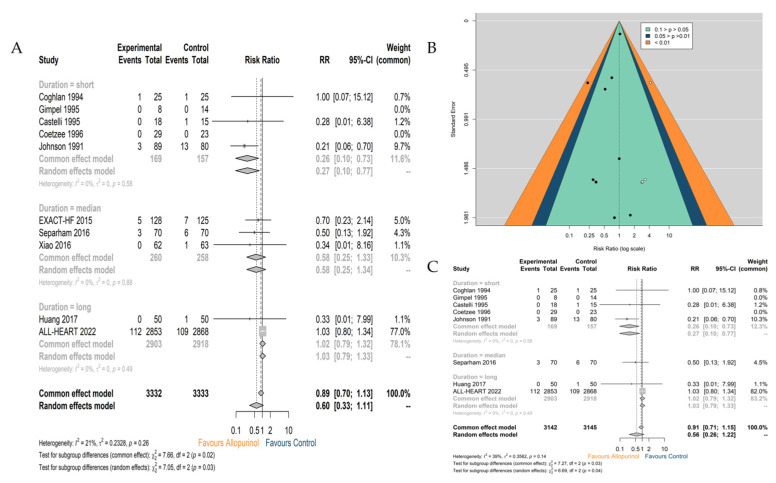
Outcome: Cardiovascular death. (**A**) Pooled analysis among all eligible patients with cardiovascular disease; (**B**) funnel plots using the trim and fill method; (**C**) pooled analysis among all subgroups with coronary artery disease. Note: Control: Placebo or usual care.

Ten studies comprising 6665 patients investigated the association of uric-lowering pharmacotherapy using allopurinol with CV death, suggesting no significant reduction in CV mortality (RR 0.60; 95% CI 0.33–1.11) (Figure 3A). The pooled analysis of five studies providing the effect of allopurinol on periprocedural CV death (≤30 days) in patients undergoing CABG suggested a lower risk for those treated with allopurinol (RR 0.27; 95%CI 0.10–0.77). The medium-term (RR 0.58; 95%CI 0.25–1.34) and long-term (RR 1.03; 95%CI 0.79–1.33) CV death rates were not reduced by allopurinol therapy. Based on the funnel plot (Figure 3B), there was no evidence of publication bias (Egger test *p*  =  0 .25). However, the use of the trim and fill method indicated the probability that studies with negative or equivocal results have not been published. The sensitivity analysis suggested that the ALL-HEART trial [12] contributed to the major heterogeneity since the result was reversed after omitting it (RR 0.43; 95% CI 0.23–0.81) (Figure S1A).

The additional subgroup analysis across patients with CAD consistently demonstrated the null effect (RR 0.56; 95% CI 0.26–1.22) (Figure 3C), and the corresponding sensitivity analysis also suggested the great influence of the ALL-HEART trial since the result was reversed after omitting it. The remaining studies, which were consistently conducted in post-ACS patients or those who underwent CABG, supported the benefit of allopurinol (omitting ALL-HEART: RR 0.34; 95% CI 0.15–0.76) (Figure S2). 

Only one eligible study investigated the effect of uric-lowering treatment using allopurinol in those with cardiovascular disease and hyperuricemia or a history of gout, reporting the failure of allopurinol to reduce CV mortality at 24 weeks (RR 0.70; 95% CI 0.23–2.14) [15]. Xiao et al. found no obvious differences between the two groups of patients with heart failure (0/62 in the allopurinol arm versus 1/63 in the usual care arm) [33]. The analysis for hypertensive individuals could not be performed owing to the lack of relevant studies.

#### 3.2.2. All-Cause Death

**Figure 4:**
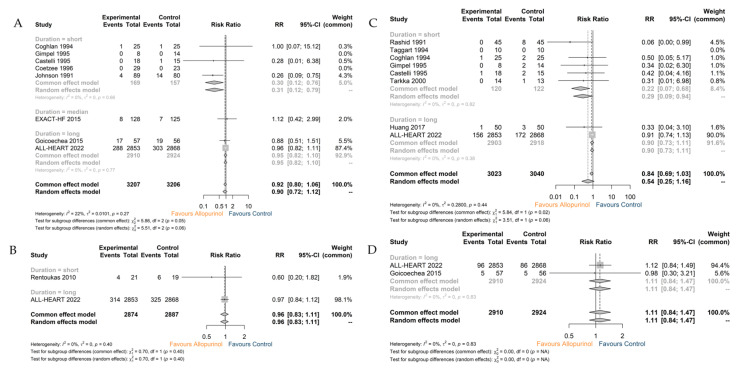
(**A**) The analysis for all-cause death; (**B**) the analysis for MACEs; (**C**) the analysis for MI; (**D**) the analysis for stroke. Note: Control: Placebo or usual care.

A total of eight studies comprising 6413 patients investigated the association of uric acid-lowering pharmacotherapy using allopurinol with all-cause death. The overall mortality rate did not significantly differ between the two groups (RR 0.90; 95% CI 0.72–1.12). A substantially reduced postoperative all-cause mortality rate was observed among the patients undergoing CABG (RR 0.31; 95% CI 0.12–0.79) (Figure 4A). Consistently, the sensitivity analysis also demonstrated the significant effect of the ALL-HEART trial on the results (Figure S1B).

#### 3.2.3. MACEs, MI and Stroke

Analysis of two studies comprising 5761 patients indicated the failure of allopurinol on MACEs despite the follow-up duration (RR 0.96; 95% CI 0.83–1.11) (Figure 4B). Consistently, the incidence of MI was also identical (RR 0.54; 95% CI 0.25–1.16). The stratified analysis indicated the remarkable effect (RR 0.29; 95% CI 0.09–0.94) of allopurinol on postoperative MI (within 30 days after revascularization) in individuals undergoing CABG [24,25,26,27,28,30] (Figure 4C). The incidence of stroke in patients receiving allopurinol therapy was not obviously reduced (RR 1.11; 95% CI 0.84–1.47) (Figure 4D). Sensitivity analysis still suggested the significant effect of the ALL-HEART trial in the production of a negative result of analysis for MI (Figure S1C).

### 3.3. Comparison 2. Allopurinol versus Febuxostat

#### 3.3.1. Cardiovascular Death and All-Cause Death

**Figure 5:**
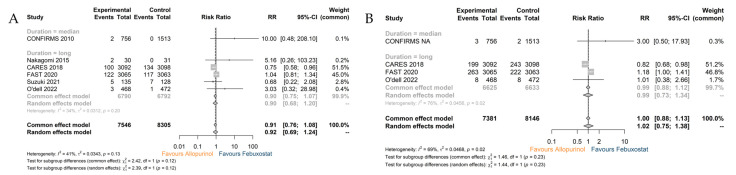
Outcome: (**A**) The analysis for cardiovascular death; (**B**) the analysis for all-cause death. Note: Control: Febuxostat.

Compared to the administration of febuxostat, allopurinol therapy did not show an additional beneficial effect on reducing CV mortality (RR 0.92; 95% CI 0.69–1.24) (Figure 5A). All-cause mortality in the two treatment groups was also identical (RR 1.02; 95% CI 0.75–1.38) (Figure 5B). The corresponding sensitivity analyses for the two outcomes did not demonstrate a significant effect of any individual study (Figure S3A,B).

#### 3.3.2. MACEs, MI, and Stroke

**Figure 6:**
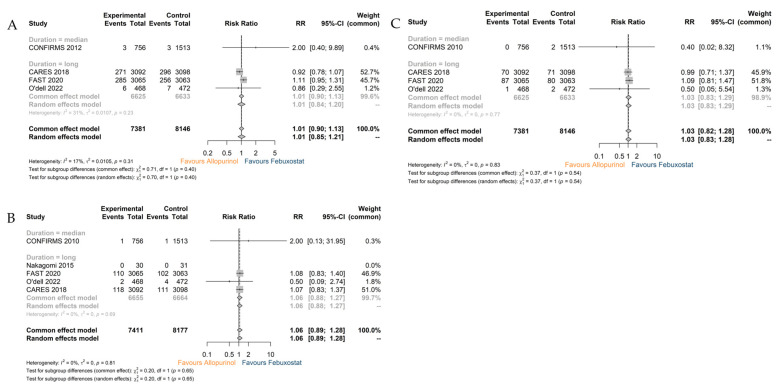
Outcome: (**A**) The analysis for MACEs; (**B**) the analysis for MI; (**C**) the analysis for stroke. Note: Control: Febuxostat.

The incidences of MACEs (RR 1.01; 95% CI 0.85–1.21), MI (RR 1.06; 95% CI 0.89–1.28), and stroke (RR 1.03; 95% CI 0.83–1.28) were all comparable (Figure 6A–C). The individual studies did not exert a critical impact on the final results of the analyses, according to the sensitivity analyses (Figure S3C,D).

## 4. Discussion

The main findings of our study are as follows. In patients with cardiovascular disease, the prescription of allopurinol did not yield significant benefits in preventing cardiovascular outcomes, including cardiovascular (CV) death, all-cause death, major adverse cardiovascular events (MACEs), myocardial infarction (MI), and stroke. However, evidence from earlier studies and small-sample investigations revealed a potential association between the initiation of allopurinol treatment and a reduction in CV mortality, overall mortality, and incidence of MI in patients undergoing CABG and those with acute coronary syndrome ACS in the short term. In the most clinically relevant active comparisons between allopurinol and febuxostat treatments, allopurinol was not found to be superior in reducing mortality and adverse cardiovascular outcomes, including CV death, MACEs, MI, and stroke. Recent narrative and systematic reviews have summarized the cardiovascular effects of allopurinol in individuals with hyperuricemia or chronic gout [7,34], but the impact in the specific cohort with cardiovascular disease (CVD) remains unclear. Van der Pol et al. [7] found that allopurinol treatment reduced the risk of a combined endpoint of cardiovascular mortality, myocardial infarction, and stroke compared with no treatment or placebo in patients with hyperuricemia (RR 0.65; 95% CI 0.46–0.91). Instead, our study focused on patients with CVD and only a few studies included patients with gout or hyperuricemia. According to studies included by us, there was no evidence of cardiovascular benefit of allopurinol in the hyperuricemia population. The EXACT-HF study [15] that was performed on patients with heart failure and hyperuricemia showed a null effect of allopurinol compared with the placebo. In the recently published ALL-HEART trial [12], the subgroup analysis according to tertiles of baseline serum uric acid concentration showed no significant effect on cardiovascular outcomes. Gao et al. [34] compared allopurinol with febuxostat in patients with chronic gout. They found that febuxostat had an advantage over allopurinol in outcomes of urgent coronary revascularization (OR 0.84; 95% CI 0.77–0.90) and stroke (OR 0.87; 95% CI 0.79–0.97). However, that difference was not found in cardiovascular mortality (OR 0.98; 95% CI 0.69–1.38). They did not conduct subgroup analysis according to the history of CVD. Our study focused on patients with CVD, in which theoretically more outcome events should happen, and it could better reflect the difference between the two drugs. In addition, our analysis included two newly published studies [18,20]. As purine is degraded in the metabolic process, the activation of xanthine oxidase (XO) results in the creation of reactive oxygen species (ROS) and a pro-inflammatory vascular state, possibly increasing the likelihood of cardiovascular disease (CVD) [35]. Allopurinol, as an inhibitor of XO, is thought to reduce oxidative stress, decrease systemic inflammation, and reverse endothelial dysfunction [36]. Prior findings from original studies and reviews suggested the potential of allopurinol in protecting against the progression of CVD by modifying vascular endothelial function [37], enhancing myocardial efficiency [38], reducing blood pressure [39], mitigating ischemia reperfusion injury [40], etc. However, these physiological endpoints are insufficient proxies for assessing clinical prognosis, necessitating further investigation to determine if allopurinol can reduce hard cardiovascular outcomes in established CVD. Contrary to our initial hypothesis, the results indicated that initiating allopurinol in patients with known CVD did not reduce primary or secondary cardiovascular outcomes. The explanation for this observation may be multifactorial. On one hand, the inherent severity and natural progression of cardiovascular disease cannot be easily reversed. On the other hand, there is a lack of clarity and ongoing debate surrounding the genuine role of uric acid in CVD. Despite being implicated by considerable research due to its pro-inflammatory and proatherogenic properties, uric acid has also been favored in the treatment of chronic vascular disease by some studies based on its potent antioxidant effects [41,42]. Given the dual biological properties, minimizing serum uric acid (SUA) levels should not be equated with maximizing clinical benefits and may even be detrimental to some extent, especially in patients without a history of hyperuricemia or gout [43]. 

However, patients undergoing coronary artery bypass grafting (CABG) or those with acute coronary syndrome (ACS) may experience potential benefits from the initial allopurinol treatment. The sensitivity analysis indicated that the ALL-HEART trial contributed to the negative association between therapy and cardiovascular (CV) death reduction across the coronary artery disease (CAD) subgroup. Unlike subjects with relatively stable ischemic heart disease in the ALL-HEART trial, patients from the other randomized control trials (RCTs) in the subgroup analysis were individuals receiving CABG or presenting with ACS [22,23,25,27,28,29,32], characterized by lower plasma antioxidant levels and more active inflammation [44]. These factors rendered the benefits of allopurinol observable in the postoperative period and acute phase of ACS. A series of studies have demonstrated that timely initiation of allopurinol treatment can effectively inhibit oxidative stress and efficiently improve endothelial function by inhibiting xanthine oxidase [45], a major contributor to vascular tissue oxidative stress and dysfunction [46]. Mellin’s experiment further discovered that only acute allopurinol treatment, rather than chronic therapy, could reduce reactive oxygen species in the left ventricle [47]. Additionally, the ability to prevent tissue damage induced by ischemia and reperfusion and facilitate left ventricular function recovery, as evidenced by some animal experiments and clinical trials, may also explain the early benefits [40,44,47]. Our analysis for all-cause death and MI consistently supported the potential short-term benefits. However, this finding should be interpreted with caution due to the obvious limitations of the original studies. The included trials with small sample sizes were mainly conducted in earlier years, thus downgrading the strength of the evidence. With advances in revascularization technology and the application of guideline-recommended pharmacotherapy in recent decades, the death rate during hospitalization has significantly decreased. Therefore, the effects observed in the earlier years of CAD treatment warrant reassessment. Further confirmation is needed to determine whether this old medicine can be used as a new trick in CAD, especially in ACS cases.

The urate-lowering agents allopurinol and febuxostat are both xanthine oxidase inhibitors and are widely prescribed in clinical practice. Becker et al. demonstrated that febuxostat exhibited greater hypouricemic activity compared to commonly used doses of allopurinol [16]. However, a series of clinical trials, including the CARES trial conducted as an FDA requirement, revealed its inferiority concerning cardiovascular events compared to allopurinol, leading to the avoidance of febuxostat in patients with a history of CVD. Nevertheless, in alignment with findings from previous studies conducted in patients with or without CVD [48], our analysis did not observe an excess of death and cardiovascular events in the febuxostat group. Reviewing these RCTs, we found that compared to patients enrolled in other trials, subjects in the CARES trial were characterized by a higher urate crystal burden and higher proportions of baseline heart failure and previous MI, all of which were predictors of poor prognosis. Furthermore, the relatively lower dose of febuxostat may also contribute to the magnitude of the difference in therapeutic effects. In fact, compared to the prescription in the CARES trial, the dosage of febuxostat used in the FAST study [11] was higher (40–80 mg/day versus 80 or 120 mg/day). The non-inferiority of febuxostat in FAST made us reconsider the ‘foe’ role defined by FDA in CVD seriously. Considering the neutral stance of previous relevant systematic reviews and the latest evidence from the FAST trial, our study does not support the superiority of allopurinol regarding cardiovascular benefits [34,49]. Our study systematically summarized the role of allopurinol in secondary cardiovascular disease prevention, and found that allopurinol may be effective in specific CVD populations. However, our work had several limitations. (1) The majority of trials included in this analysis had small sample sizes, which may suggest that they were underpowered to detect differences between different interventions. (2) The variation in the time when the trials were conducted, ranging from 1990 to 2022, indicated that significant clinical heterogeneity existed among the enrolled subjects. Evidence from studies in the earlier years should be further elucidated. (3) Due to the lack of original studies related to specific patient populations, such as those with hypertension and heart failure, we were unable to conduct corresponding subgroup analyses. (4) The therapeutic effect and prognosis in CVD patients with hyperuricemia or gout differed significantly from those without disorders of uric acid metabolism. Only a small number of trials included in our study focused on the therapeutic effect of allopurinol in patients with hyperuricemia or gout. Consequently, the extent to which our findings are generalizable to various CVD patients remains unclear.

## 5. Conclusions

Allopurinol could not reduce cardiovascular (CV) death or major adverse CV outcomes significantly in patients with existing cardiovascular diseases. Allopurinol is not recommended for secondary cardiovascular disease prevention in patients without a history of hyperuricemia or gout. Given the limitations of the original studies, the potential advantages of allopurinol observed in patients undergoing CABG or presenting with ACS necessitate further confirmation through subsequent RCTs. In the comparisons between allopurinol and febuxostat, our analysis failed to uncover any marked superiority of allopurinol in reducing the risk of adverse cardiovascular incidents.

## Figures and Tables

**Table 1:** Key features of the included trials.

Trial Acronym	Author	Year	Trial Size	Key Inclusion Criteria	Active Treatment	Comparator	Multi-Center	Open-Label	Follow-Up (Median)
-	Johnson [23]	1991	169	requiring CABG	allopurinol 200–400 mg	placebo	No	No	30 days
-	Rashid [24]	1991	90	requiring CABG	allopurinol 600 mg	usual care	No	Yes	perioperative
-	Coghlan [25]	1994	50	requiring CABG	allopurinol 300 mg	placebo	No	No	perioperative
-	Taggart [26]	1994	20	requiring CABG	allopurinol 1200 mg	usual care	No	Yes	perioperative
-	Castelli [27]	1995	33	requiring CABG	allopurinol 200 mg	usual care	No	No	perioperative
-	Gimpel [28]	1995	22	requiring CABG	allopurinol 300 mg	usual care	No	Yes	perioperative
-	Coetzee [29]	1996	52	requiring CABG	allopurinol 800 mg	usual care	No	No	perioperative
-	Tarkka [30]	2000	27	requiring CABG	allopurinol 800 mg	placebo	No	No	perioperative
-	Rentoukas [31]	2010	40	acute myocardial infarction undergoing primary percutaneous coronary intervention	allopurinol (loading dose of 400 mg and maintenance dose of 100 mg)	placebo	Yes	No	30 days
EXACT-HF	Givertz [15]	2015	253	heart failure with hyperuricemia	allopurinol 300–600 mg	placebo	Yes	No	24 weeks
-	Goicoechea [21]	2015	113	patients with eGFRs < 60 mL/min/1.73 m2, stable clinical condition, and stable kidney function	allopurinol 100 mg	standard treatment	No	No	84 months
-	Separham [32]	2016	140	STEMI undergoing thrombolytic therapy	allopurinol (loading dose of 400 mg and maintenance dose of 100 mg)	placebo	No	No	6 months
-	Xiao [33]	2016	125	chronic heart failure	allopurinol 300 mg	usual care	No	Yes	9.6 months
-	Huang [22]	2017	100	acute coronary syndrome	allopurinol 600 mg	usual care	No	Yes	2 years
ALL-HEART	Mackenzie [12]	2022	5721	ischemic heart disease	allopurinol 600 mg	usual care	Yes	Yes	4.8 years
CONFIRMS	Becker [16]	2010	2269	gout and serum urate ≥ 8.0 mg/dL	allopurinol 200 or 300 mg	febuxostat 40 mg or 80 mg	Yes	No	6 months
CARES	White [19]	2018	6190	gout and a history of major cardiovascular disease	allopurinol 100–600 mg	febuxostat 40–80 mg	Yes	No	968 days in the febuxostat group and 942 days in the allopurinol group
FAST	Mackenzie [11]	2020	6128	gout, aged 60 years or older, with cardiovascular risk factor	allopurinol 100–900 mg	febuxostat 80 or 120 mg	Yes	Yes	1467 days
-	Suzuki [20]	2021	263	chronic heart failure with hyperuricemia	allopurinol initial dose 200 mg	febuxostat initial dose 10 mg	Yes	No	3 years
-	Nakagomi [17]	2015	61	chronic heart failure with hyperuricemia	allopurinol 100–300 mg	febuxostat 10–40 mg	No	Yes	23 months
-	O’Dell [18]	2022	940	gout	allopurinol 200–800 mg	febuxostat 40–120 mg	Yes	No	72 weeks

## Data Availability

The analytic dataset is available upon request by contacting the corresponding author.

## Supplementary Materials

The following supporting information can be downloaded at: https://www.mdpi.com/article/10.3390/jcdd10090379/s1, Search strategy; Changes from protocol; Figure S1: Sensitivity analysis for the pooled analysis comparing allopurinol with placebo/usual care; Figure S2: Sensitivity analysis for the pooled analysis for CV death comparing allopurinol with placebo/usual care among the sub-cohort with coronary artery disease or ischemic heart disease; Figure S3: Sensitivity analysis for the pooled analysis comparing allopurinol with febuxostat; Table S1: Baseline characteristics of patients enrolled; Table S2: The risk-of-bias table for individual studies.
