# Supplementary material for: Allopurinol for Secondary Prevention in Patients with Cardiovascular Disease: A Systematic Review and Meta-Analysis of Randomized Controlled Trials
Source: J Cardiovasc Dev Dis. 2023 Sep 4;10(9):379. doi: 10.3390/jcdd10090379 (PMC10532321; doi:10.3390/jcdd10090379)
Supplement: Supplementary file 1 [file jcdd-10-00379-s001.zip › jcdd-2481542-supplementary.pdf]

Supplemental appendix

Supplement to:

Allopurinol for Secondary Prevention in Patients with Cardiovascular Disease: A Systematic Review and Meta-analysis of Randomized Controlled Trials

1. Search strategy

	strategy	hits
PubMed		
#1	Allopurinol[Mesh] OR allopurinol[tiab] OR Uribenz[tiab] OR Allopurin[tiab] OR Allorin[tiab] OR Allpargin[tiab] OR Allural[tiab] OR Pan Quimica[tiab] OR Apulonga[tiab] OR Apurin[tiab] OR Atisuril[tiab] OR Bleminol[tiab] OR Caplenal[tiab] OR Capurate[tiab] OR Cellidrin[tiab] OR Embarin[tiab] OR Suspendol[tiab] OR Foligan[tiab] OR Hamarin[tiab] OR Lopurin[tiab] OR Lysuron[tiab] OR Jenapurinol[tiab] OR Milurit[tiab] OR Milurite[tiab] OR Novopurol[tiab] OR Uripurinol[tiab] OR Urosin[tiab] OR Urtias[tiab] OR Xanthomax[tiab] OR Uridocid[tiab] OR Xanturic[tiab] OR Zygout[tiab] OR Zyloprim[tiab] OR Zyloric[tiab] OR Pureduct[tiab] OR Purinol[tiab] OR Progout[tiab] OR Remid[tiab] OR Rimapurinol[tiab] OR Roucol[tiab] OR Tipuric[tiab] OR Allohexal[tiab] OR Allohexan[tiab] OR Alloprin[tiab]	11,226
#2	"Cardiovascular Diseases"[Mesh] OR "cardiovascular disease*"[tiab] OR CVD[tiab] OR "Heart Diseases"[Mesh] OR "heart disease*"[tiab] OR "heart disorder*"[tiab] OR "cardiac disease*"[tiab] OR "cardiac disorder*"[tiab] OR "heart attack"[tiab] OR "heart*"[tiab] OR "cardiac"[tiab] OR "Hypertension"[Mesh] OR "Blood Pressure*" [tiab] OR "Hypertension"[tiab] OR "Hypersensitive" [tiab] OR "Pulmonary Heart Disease"[Mesh] OR "Pulmonary Heart Disease*"[tiab] OR "Cor Pulmonale"[tiab] OR "Myocardial Ischemia"[Mesh] OR "myocardial ischemia"[tiab] OR "ischemic heart disease"[tiab] OR "Coronary Artery Disease"[Mesh] OR "coronary artery disease"[tiab] OR "coronary heart disease"[tiab] OR CAD[tiab] OR "coronary disease"[tiab] OR "myocardial infarction"[tiab] OR "angina"[tiab] OR "acute coronary syndrome"[tiab] OR "coronary"[tiab] OR "Heart Failure"[Mesh] OR "Heart Failure"[tiab] OR "Cardiac Failure"[tiab] OR "Heart Decompensation"[tiab] OR "Myocardial Failure"[tiab] OR "Congestive Heart Failure"[tiab] OR "Cerebrovascular Disorders"[Mesh] OR "Cerebrovascular Disorder*"[tiab] OR "Intracranial Vascular Disease*"[tiab] OR "Intracranial Vascular Disorder*"[tiab] OR "Cerebrovascular Disease*"[tiab] OR "Brain Vascular Disorder*"[tiab] OR "Brain Vascular Disease*"[tiab] OR "Cerebrovascular Occlusion*"[tiab] OR "Cerebrovascular Insufficiency"[tiab] OR "Brain	3,863,631

	Ischemia”[Mesh] OR “Brain Ischemia*”[tiab] OR “Ischemic Encephalopath*”[tiab] OR “Cerebral Ischemia*”[tiab] OR “Brain infarction”[tiab] OR “Cerebral infarction”[tiab] OR “Brain Stem infarction”[tiab] OR “Transient ischemic attack”[tiab] OR TIA[tiab] OR “Stroke”[Mesh] OR “Stroke”[tiab] OR “Cerebrovascular accident”[tiab] OR CVA[tiab] OR "Arteriosclerosis"[Mesh] OR “Arteriosclerosis”[tiab] OR "Atherosclerosis"[tiab] OR “Arterioscleroses”[tiab] OR ASCVD[tiab] OR "Peripheral Arterial Disease"[Mesh] OR “Peripheral Arterial Disease*”[tiab] OR PAD[tiab] OR “Intermittent Claudication”[tiab] OR “Fontaine”[tiab] OR MACE[tiab] OR “major adverse cardiovascular events”[tiab] OR “cardiovascular events”[tiab] OR “cardiac events”[tiab] OR “cardiac death”[tiab] OR “cardiac mortality”[tiab] OR “cardiovascular death”[tiab] OR “cardiovascular mortality”[tiab]	
#3	(“randomized controlled trial”[pt] OR “controlled clinical trial”[pt] OR randomized[tiab] OR placebo[tiab] OR “drug therapy”[sh] OR randomly[tiab] OR trial[tiab] OR groups[tiab]) NOT (animals [mh] NOT humans [mh])	4,935,960
#4	#1 AND #2 AND #3	872
Embase		
#1	‘allopurinol’/exp OR ‘allopurinol’:ab,ti,kw OR ‘uribenz’:ab,ti,kw OR ‘allopurin’:ab,ti,kw OR ‘allorin’:ab,ti,kw OR ‘allpargin’:ab,ti,kw OR ‘allural’:ab,ti,kw OR ‘pan quimica’:ab,ti,kw OR ‘apulonga’:ab,ti,kw OR ‘apurin’:ab,ti,kw OR ‘atisuril’:ab,ti,kw OR ‘bleminol’:ab,ti,kw OR ‘caplenal’:ab,ti,kw OR ‘capurate’:ab,ti,kw OR ‘cellidrin’:ab,ti,kw OR ‘embarin’:ab,ti,kw OR ‘suspendol’:ab,ti,kw OR ‘foligan’:ab,ti,kw OR ‘hamarin’:ab,ti,kw OR ‘lopurin’:ab,ti,kw OR ‘lysuron’:ab,ti,kw OR ‘jenapurinol’:ab,ti,kw OR ‘milurit’:ab,ti,kw OR ‘milurite’:ab,ti,kw OR ‘novopurol’:ab,ti,kw OR ‘uripurinol’:ab,ti,kw OR ‘urosin’:ab,ti,kw OR ‘urtias’:ab,ti,kw OR ‘xanthomax’:ab,ti,kw OR ‘uridocid’:ab,ti,kw OR ‘xanturic’:ab,ti,kw OR ‘zygout’:ab,ti,kw OR ‘zyloprim’:ab,ti,kw OR ‘zyloric’:ab,ti,kw OR ‘pureduct’:ab,ti,kw OR ‘purinol’:ab,ti,kw OR ‘progout’:ab,ti,kw OR ‘remid’:ab,ti,kw OR ‘rimapurinol’:ab,ti,kw OR ‘roucol’:ab,ti,kw OR ‘tipuric’:ab,ti,kw OR ‘allohexal’:ab,ti,kw OR ‘allohexan’:ab,ti,kw OR ‘alloprin’:ab,ti,kw	27,457
#2	'cardiovascular disease'/exp OR ‘cardiovascular disease*’:ab,ti,kw OR ‘cvd’:ab,ti,kw OR 'heart disease'/exp OR ‘heart disease*’:ab,ti,kw OR ‘heart disorder*’:ab,ti,kw OR ‘cardiac disease*’:ab,ti,kw OR ‘cardiac disorder*’:ab,ti,kw OR ‘heart attack’:ab,ti,kw OR ‘heart*’:ab,ti,kw OR ‘cardiac’:ab,ti,kw OR 'hypertension'/exp OR ‘Blood Pressure*’:ab,ti,kw OR ‘Hypertension’:ab,ti,kw OR ‘Hypersensitive’:ab,ti,kw OR 'cor pulmonale'/exp OR ‘Pulmonary Heart Disease*’:ab,ti,kw OR ‘Cor Pulmonale’:ab,ti,kw OR ‘ischemic heart disease’/exp OR ‘ischemic heart disease’:ab,ti,kw OR ‘Myocardial Ischemia’:ab,ti,kw OR ‘coronary artery disease’/exp OR ‘coronary artery disease’:ab,ti,kw OR CAD:ab,ti,kw OR ‘coronary heart disease’:ab,ti,kw OR ‘coronary disease’:ab,ti,kw OR ‘myocardial infarction’:ab,ti,kw OR ‘angina’:ab,ti,kw OR ‘acute coronary	6,238,338

	syndrome’:ab,ti,kw OR ‘coronary’:ab,ti,kw OR 'heart failure'/exp OR ‘Heart Failure’:ab,ti,kw OR ‘Cardiac Failure’:ab,ti,kw OR ‘Heart Decompensation’:ab,ti,kw OR ‘Myocardial Failure’:ab,ti,kw OR ‘Congestive Heart Failure’:ab,ti,kw OR 'cerebrovascular disease'/exp OR ‘Cerebrovascular Disorder*’:ab,ti,kw OR ‘Intracranial Vascular Disease*’:ab,ti,kw OR ‘Cerebrovascular Disease*’:ab,ti,kw OR ‘Brain Vascular Disorder*’:ab,ti,kw OR ‘Brain Vascular Disease*’:ab,ti,kw OR ‘Cerebrovascular Occlusion*’:ab,ti,kw OR ‘Cerebrovascular Insufficiency’:ab,ti,kw OR ‘brain ischemia’/exp OR ‘Brain Ischemia’:ab,ti,kw OR ‘Ischemic Encephalopath*’:ab,ti,kw OR ‘Cerebral Ischemia*’:ab,ti,kw OR ‘Brain infarction’:ab,ti,kw OR ‘Cerebral infarction’:ab,ti,kw OR ‘Brain Stem infarction’:ab,ti,kw OR ‘Transient ischemic attack’:ab,ti,kw OR TIA:ab,ti,kw OR ‘cerebrovascular accident’/exp OR ‘Stroke’:ab,ti,kw OR ‘Cerebrovascular accident’:ab,ti,kw OR CVA:ab,ti,kw OR ‘Atherosclerosis’/exp OR ‘Arteriosclerosis’:ab,ti,kw OR ‘Atherosclerosis’:ab,ti,kw OR ‘Arterioscleroses’:ab,ti,kw OR ASCVD:ab,ti,kw OR 'peripheral arterial disease'/exp OR ‘Peripheral Arterial Disease*’:ab,ti,kw OR PAD:ab,ti,kw OR ‘Intermittent Claudication’:ab,ti,kw OR ‘Fontaine’:ab,ti,kw OR ‘major adverse cardiovascular events’:ab,ti,kw OR MACE:ab,ti,kw OR ‘cardiovascular events’:ab,ti,kw OR ‘cardiac events’:ab,ti,kw OR ‘cardiovascular death’:ab,ti,kw OR ‘cardiovascular mortality’:ab,ti,kw OR ‘cardiac death’:ab,ti,kw OR ‘cardiac mortality’:ab,ti,kw	
#3	((‘randomized controlled trial’/de OR ‘controlled clinical trial’/de OR random*:ti,ab,tt OR ‘randomization’/de OR ‘intermethod comparison’/de OR placebo:ti,ab,tt OR (compare:ti,tt OR compared:ti,tt OR comparison:ti,tt) OR ((evaluated:ab OR evaluate:ab OR evaluating:ab OR assessed:ab OR assess:ab) AND (compare:ab OR compared:ab OR comparing:ab OR comparison:ab)) OR (open NEXT/1 label):ti,ab,tt OR ((double OR single OR doubly OR singly) NEXT/1 (blind OR blinded OR blindly)):ti,ab,tt OR ‘double blind procedure’/de OR (parallel NEXT/1 group*):ti,ab,tt OR (crossover:ti,ab,tt OR ‘cross over’:ti,ab,tt) OR ((assign* OR match OR matched OR allocation) NEAR/6 (alternate OR group OR groups OR intervention OR interventions OR patient OR patients OR subject OR subjects OR participant OR participants)):ti,ab,tt OR (assigned:ti,ab,tt OR allocated:ti,ab,tt) OR (controlled NEAR/8 (study OR design OR trial)):ti,ab,tt OR (volunteer:ti,ab,tt OR volunteers:ti,ab,tt) OR ‘human experiment’/de OR Trial:ti,tt) NOT ((((random* NEXT/1 sampl* NEAR/8 (‘cross section*’ OR questionnaire* OR survey OR surveys OR database or databases)):ti,ab,tt) NOT (‘comparative study’/de OR ‘controlled study’/de OR ‘randomised controlled’:ti,ab,tt OR ‘randomized controlled’:ti,ab,tt OR ‘randomly assigned’:ti,ab,tt)) OR (‘cross-sectional study’/de NOT (‘randomized controlled trial’/de OR ‘controlled clinical study’/de OR ‘controlled study’/de OR ‘randomised controlled’:ti,ab,tt OR ‘randomized controlled’:ti,ab,tt OR ‘control group’:ti,ab,tt OR ‘control groups’:ti,ab,tt)) OR (‘case control*’:ti,ab,tt AND random*:ti,ab,tt NOT	2,071,062

	('randomised controlled':ti,ab,tt OR 'randomized controlled':ti,ab,tt)) OR ('systematic review':ti,tt NOT (trial:ti,tt OR study:ti,tt)) OR (nonrandom*:ti,ab,tt NOT random*:ti,ab,tt) OR 'random field*':ti,ab,tt OR ('random cluster' NEAR/4 sampl*):ti,ab,tt OR (review:ab AND review:it NOT trial:ti,tt) OR ('we searched':ab AND (review:ti,tt OR review:it)) OR 'update review':ab OR (databases NEAR/5 searched):ab OR ((rat:ti,tt OR rats:ti,tt OR mouse:ti,tt OR mice:ti,tt OR swine:ti,tt OR porcine:ti,tt OR murine:ti,tt OR sheep:ti,tt OR lambs:ti,tt OR pigs:ti,tt OR piglets:ti,tt OR rabbit:ti,tt OR rabbits:ti,tt OR cat:ti,tt OR cats:ti,tt OR dog:ti,tt OR dogs:ti,tt OR cattle:ti,tt OR bovine:ti,tt OR monkey:ti,tt OR monkeys:ti,tt OR trout:ti,tt OR marmoset*:ti,tt) AND 'animal experiment'/de) OR ('animal experiment'/de NOT ('human experiment'/de OR 'human'/de)))) NOT [medline]/lim	
#4	#1 AND #2 AND #3	475
Cochrane		
#1	MeSH descriptor: [Allopurinol] explode all trees	664
#2	(allopurinol OR Uribenz OR Allopurin OR Allorin OR Allpargin OR Allural OR Pan Quimica OR Apulonga OR Apurin OR Atisuril OR Bleminol OR Caplenal OR Capurate OR Cellidrin OR Embarin OR Suspendol OR Foligan OR Hamarin OR Lopurin OR Lysuron OR Jenapurinol OR Milurit OR Milurite OR Novopurol OR Uripurinol OR Urosin OR Urtias OR Xanthomax OR Uridocid OR Xanturic OR Zygout OR Zyloprim OR Zyloric OR Pureduct OR Purinol OR Progout OR Remid OR Rimapurinol OR Roucol OR Tipuric OR Allohexal OR Allohexan OR Alloprin):ti,ab,kw	1,441
#3	#1 OR #2	1,441
#4	MeSH descriptor: [Cardiovascular Diseases] explode all trees	134,950
#5	MeSH descriptor: [Heart Diseases] explode all trees	64,900
#6	MeSH descriptor: [Hypertension] explode all trees	22,327
#7	MeSH descriptor: [Pulmonary Heart Disease] explode all trees	75
#8	MeSH descriptor: [Myocardial Ischemia] explode all trees	34,411
#9	MeSH descriptor: [Coronary Artery Disease] explode all trees	8,182
#10	MeSH descriptor: [Heart Failure] explode all trees	12,328
#11	MeSH descriptor: [Cerebrovascular Disorders] explode all trees	20,844
#12	MeSH descriptor: [Brain Ischemia] explode all trees	4,836
#13	MeSH descriptor: [Stroke] explode all trees	14,102
#14	MeSH descriptor: [Arteriosclerosis] explode all trees	12,642
#15	MeSH descriptor: [Peripheral Arterial Disease] explode all trees	2,238
#16	("cardiovascular disease*" OR CVD OR "heart disease*" OR "heart disorder*" OR "cardiac disease*" OR "cardiac disorder*" OR "heart attack" OR "heart*" OR "cardiac" OR "Blood Pressure*" OR "Hypertension" OR "Hypersensitive" OR "Pulmonary Heart Disease*" OR "Cor Pulmonale" OR "myocardial ischemia" OR "ischemic heart disease" OR "coronary artery disease" OR "coronary heart disease" OR CAD OR "coronary disease" OR "myocardial infarction" OR "angina" OR "acute coronary syndrome" OR "coronary" OR "Heart Failure" OR	370,189

	“Cardiac Failure” OR “Heart Decompensation” OR “Myocardial Failure” OR “Congestive Heart Failure” OR “Cerebrovascular Disorder*” OR “Intracranial Vascular Disease*” OR “Intracranial Vascular Disorder*” OR “Cerebrovascular Disease*” OR “Brain Vascular Disorder*” OR “Brain Vascular Disease*” OR “Cerebrovascular Occlusion*” OR “Cerebrovascular Insufficiency” OR “Brain Ischemia*” OR “Ischemic Encephalopath*” OR “Cerebral Ischemia*” OR “Brain infarction” OR “Cerebral infarction” OR “Brain Stem infarction” OR “Transient ischemic attack” OR TIA OR “Stroke” OR “Cerebrovascular accident” OR CVA OR “Arteriosclerosis” OR "Atherosclerosis" OR “Arterioscleroses” OR ASCVD OR “Peripheral Arterial Disease*” OR PAD OR “Intermittent Claudication” OR “Fontaine” OR MACE OR “major adverse cardiovascular events” OR “cardiovascular events” OR “cardiac events” OR “cardiac death” OR “cardiac mortality” OR “cardiovascular death” OR “cardiovascular mortality”):ti,ab,kw	
#17	#4 OR #5 OR #6 OR #7 OR #8 OR #9 OR #10 OR #11 OR #12 OR #13 OR #14 OR #15 OR #16	394,768
#18	#3 AND #17	549

2. Changes from protocol

We initially defined cardiovascular disease as the compositions including cerebrovascular disease and peripheral vascular disease, but when we reconsider the definition later we hold that the heterogeneity among these diseases was so significant that we finally decided to limit this definition to the coronary artery disease, any ischemic heart disease, hypertension, and heart failure. They are common cardiovascular disease and the progress have been supposed to be associated with serum uric acid. In addition, we supplemented some subgroup analyses to provide more evidence-based information. Sensitivity analysis was performed to explore underlying causes of heterogeneity.

3. Supplementary tables

Table S1: Baseline characteristics of patients enrolled.

Acronym or author	Age (years)	Females (%)	Body-mass index (kg/m²)	Diabetes (%)	Hypertension (%)	eGFR<60 mL/min/1.73m^2 (%)	Myocardial infarction (%)	Stroke (%)	Dyslipidemia (%)	Heart failure (%)	Serum urate (mg/dL)	Peripheral arterial disease (%)
Johnson1991	60.1	16.0	-	25.4	-	26.6	-	-	-	-	-	-
Rashid1991	62.2	24.4	-	-	-	0.0	-	-	-	-	-	-
Coghlan1994	58.0	16.0	-	14.0	-	-	34.0	-	-	-	-	-
Taggart1994	60.0	0.0	-	-	-	-	-	-	-	-	-	-
Castelli1995	61.2	6.1	-	-	-	-	78.8	-	-	100.0	-	-
Gimpel1995	58.5	22.7	-	-	-	-	50.0	-	-	-	-	-

Coetzee1996	59.4	9.6	-	-	-	-	-	-	-	-	-	-
Tarkka2000	60.9	0.0	-	22.2	-	-	51.9	-	-	-	-	-
CONFIRMS2010	52.8	5.6	32.8	13.8	-	17.7	-	-	41.5	-	9.6	-
Rentoukas2010	64.2	27.5	-	22.5	25.0	-	100.0	-	-	-	6.9	-
EXACT-HF2015	63.0	17.8	32.3	54.5	78.3	-	-	-	-	100.0	11.0	-
Goicoechea2015	71.8	-	-	37.2	-	100.0	-	-	-	-	7.6	5.3
Nakagomi2015	70.5	34.4	23.4	34.4	93.4	-	-	-	96.7	100.0	-	-
Separham2016	60.7	22.1	-	25.7	42.1	-	100.0	-	15.0	-	5.2	-
Xiao2016	51.9	26.4	-	-	-	-	-	-	-	-	-	-
Huang2017	-	-	-	-	-	-	-	-	-	-	8.9	-
CARES2018	64.5	16.1	33.5	-	92.3	52.8	39.2	14.1	86.9	20.2	8.7	12.7
FAST2020	70.9	14.7	31.1	22.5	78.1	-	10.7	5.0	-	4.7	5.0	4.8
Suzuki2021	71.0	33.5	-	29.3	72.6	-	-	-	57.8	100.0	8.6	-
ALL-HEART2022	72.0	24.4	28.8	21.7	57.5	9.4	47.3	4.0	63.3	5.2	5.7	7.5
O'Dell2022	62.1	1.6	33.7	33.3	76.4	37.3	-	-	-	-	8.5	-

Table S2: The risk of bias table for individual study.

**Study**	**D1**	**D2**	**D3**	**D4**	**D5**	**Overall**
Johnson1991	Low	Low	Low	Low	Some concerns	Some concerns
Rashid1991	Low	Low	Low	Low	Some concerns	Some concerns
Coghlan1994	Low	Low	Low	Low	Some concerns	Some concerns
Taggart1994	Low	Low	Low	Low	Some concerns	Some concerns
Castelli1995	Some concerns	Low	Low	Low	Some concerns	Some concerns
Gimpel1995	Some concerns	Low	Low	Low	Some concerns	Some concerns
Coetzee1996	Low	Low	Low	Low	Some concerns	Some concerns
Tarkka2000	Some concerns	Low	Low	Low	Some concerns	Some concerns
CONFIRMS2010	Low	Low	Some concerns	Low	Some concerns	Some concerns
Rentoukas2010	Some concerns	Low	Low	Low	Some concerns	Some concerns
Taheraghdam2014	Low	Low	Low	Low	Some concerns	Some concerns
EXACT-HF2015	Low	Low	Low	Low	Low	Low
Goicoechea2015	Low	High	Some concerns	Low	Low	High
Nakagomi2015	Low	Low	Low	Low	Some concerns	Some concerns
Separham2016	Low	Low	Low	Low	Some concerns	Some concerns
Xiao2016	Some concerns	Some concerns	Low	Low	Some concerns	Some concerns
Huang2017	Some concerns	Some concerns	High	Low	Some concerns	High
CARES2018	Low	Low	Some concerns	Low	Low	Some concerns

FAST2020	Low	Some concerns	Low	Low	Low	Some concerns
Suzuki2021	Low	Some concerns	High	Low	Low	High
ALL-HEART2022	Low	Some concerns	Low	Low	Low	Some concerns
O'Dell2022	Low	Low	Some concerns	Low	Low	Some concerns

D1 Randomization process
D2 Deviations from the intended interventions
D3 Missing outcome data
D4 Measurement of the outcome
D5 Selection of the reported result

4. Supplementary figures

4.1 sensitivity analyses

3.1.1 Comparison 1. Allopurinol vs placebo/usual care

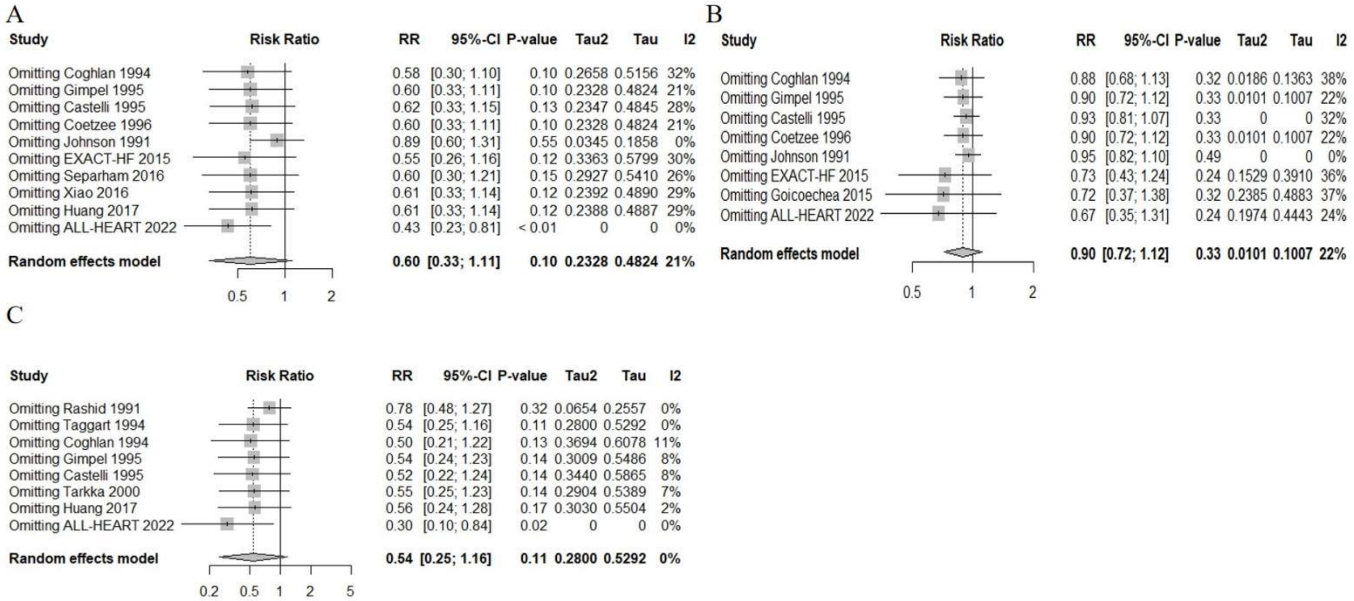

Figure S1. Sensitivity analysis for the pooled analysis comparing allopurinol with placebo/usual care.
A) Cardiovascular death; B) All-cause death; C) MI

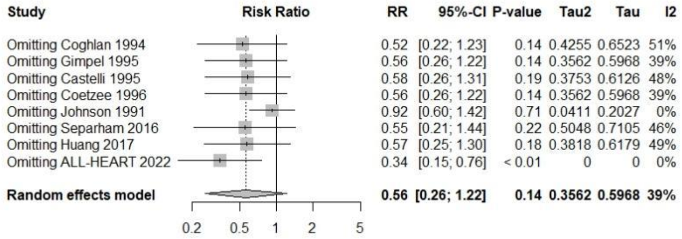

Figure S2. Sensitivity analysis for the pooled analysis for CV death comparing allopurinol with placebo/usual care among the sub-cohort with coronary artery disease or ischemic heart disease.

3.1.2 Comparison 2. Allopurinol vs febuxostat

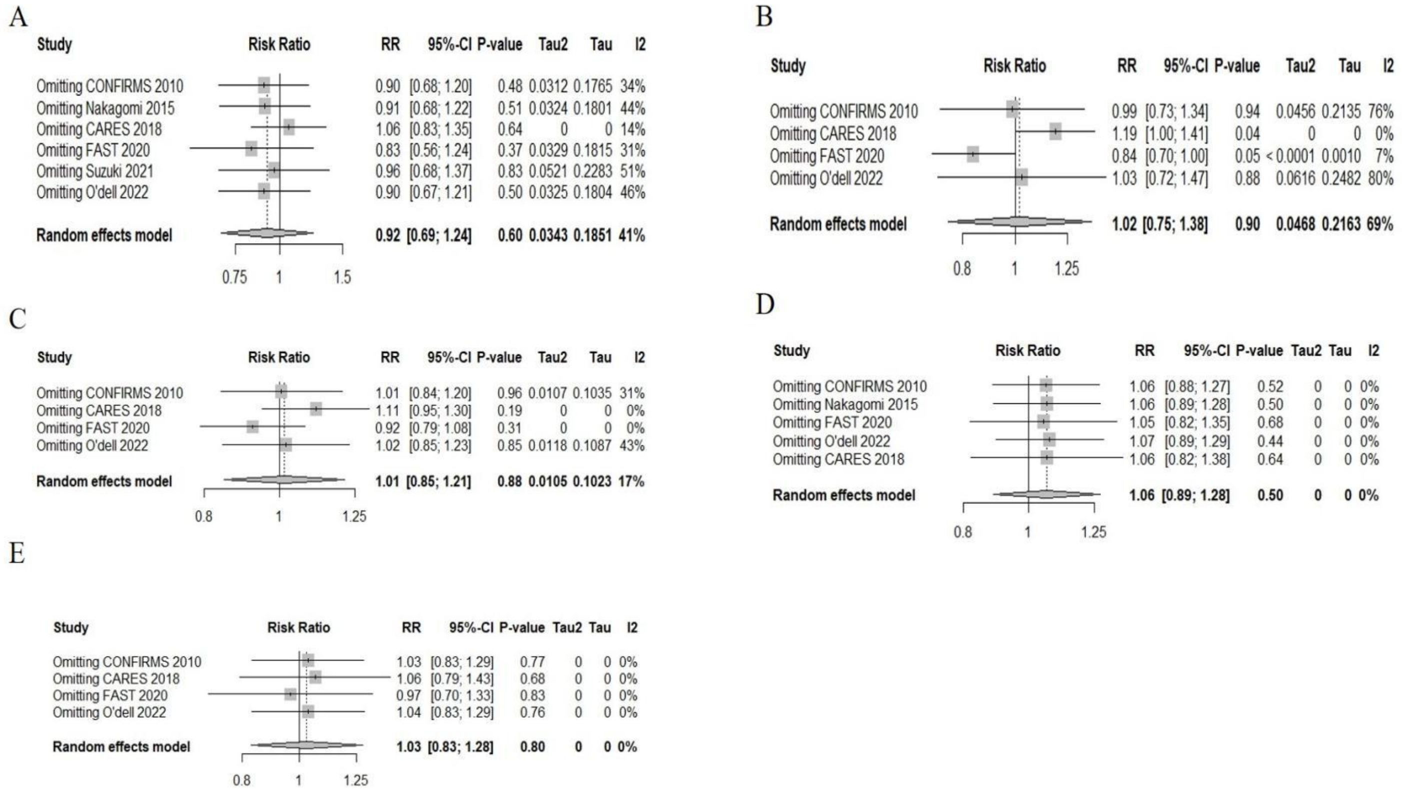

Figure S3. Sensitivity analysis for the pooled analysis comparing allopurinol with febuxostat.
A) Cardiovascular death; B) All-cause death; C) MACEs; D) MI; E) stroke
